# Spatiotemporal variability of the association between greenspace exposure and depression in older adults in South Korea

**DOI:** 10.1186/s12889-024-19952-2

**Published:** 2024-09-19

**Authors:** Eun-Hye Yoo, Jin-Young Min, Baek-Yong Choi, Seung-Woo Ryoo, Kyoung-Bok Min, John E. Roberts

**Affiliations:** 1 Department of Geography, University of Buffalo, The State University of New York, NY, USA; 2 Veterans Medical Research Institute, Veterans Health Service Medical Center, Seoul, Republic of Korea; 3 Department of Preventive Medicine, College of Medicine, Seoul National University, 103, Daehak-ro, Jongno-gu, Seoul, Republic of Korea; 4 Institute of Health Policy and Management, Medical Research Center, Seoul National University, Seoul, Republic of Korea; 5 Department of Psychology, University of Buffalo, The State University of New York, NY, USA

**Keywords:** Greenspace, Mental health, Seasonality, Spatial resolution, Older adults

## Abstract

**Background:**

A number of studies based on young to middle aged adult and child samples have found that exposure to greenspace and bluespace can have a positive impact on mental health and well-being. However, there is limited research among older adults and the extant studies have provided mixed results. The present study was designed to examine how the association between these forms of exposure and depressive symptoms among older adults varies as a function of different spatially and temporally resolved exposure metrics.

**Methods:**

The sample consisted of 617 individuals (46.19% female) aged ≥ 60 years of age. Depressive symptoms were measured using the 10-item Center for Epidemiological Studies Depression Scale (CES-D). Individuals’ greenspace exposure was quantified using spatially and temporally resolved metrics, including monthly and annual averaged satellite-derived normalized difference vegetation index (NDVI) across multiple buffer distances (250 m to 2,000 m) centered at participants’ home address. We also quantified exposure to blue-greenspace from a highly detailed land use and land cover dataset. A multivariable logistic regression model assessed the association between greenspace and blue-greenspace exposure and depressive symptoms, adjusting for age, sex, income, education, marital status, current smoking, alcohol status, medical conditions, temperature, crime rate, population density, and per capita park area.

**Results:**

We found a significant association between exposures to greenspace and blue-greenspace and depressive symptoms (CES-D cutoff ≥ 4) among older adults. After adjusting for confounding variables, the odds of depressive symptoms were significantly decreased by an IQR increment in residential exposure to greenspace [odds ratio (OR) = 0.67; 95% confidence interval (95% CI), 0.49 ~ 0.91] and blue-greenspace (OR = 0.59; 95% CI, 0.41 ~ 0.84) measured nearby their home address (i.e., as close as 250 m). When stratified by household income level, the association was only significant among low-income individuals. We also found temporal variation in the association between depressive symptoms and monthly NDVI-based greenspace exposure, in which the odds of depressive symptoms were the lowest for greenspace in cold months (i.e., January, February, and March).

**Conclusions:**

Our findings suggest that neighborhood greenspace may serve as a protective factor against depression among older adults, but the benefits may depend on the spatial and temporal context. More investigation is needed to replicate our findings on the spatial and temporal variations of greenspace exposure metrics and their effects on depressive symptoms.

**Supplementary Information:**

The online version contains supplementary material available at 10.1186/s12889-024-19952-2.

## Background

For the aging population, depression is a common mental health problem that is associated with increased risk for cardiovascular disease, dementia, and all-cause and cause-specific mortality [48]. Identifying environmental and behavioral factors that are associated with depressive symptoms in the elderly is important because modification of these factors potentially could reduce or prevent depression. Greenspace exposure has recently garnered interest in terms of its potential to promote mental health [10] and a number of pathways linking greenspace and health outcomes have been proposed, including decreasing exposure to air pollution, noise and heat, (2) attention restoration and stress recovery, and (3) promoting social cohesion and physical activity [28].

Several studies have shown a positive association between greenspace and mental health [10]; Fong et al., [14, 15, 30]. These studies quantified the amount and quality of greenspace exposure using various metrics. For example, greenspace accessibility of different subgroups is quantified by the proximity of parks and forests to residential areas or population centers. Feng. et al. (2022) showed that high quality of greenspace (i.e., perceived greenspace quality) is associated with good mental health in adolescents [13]. Lastly, landcover classification and normalized difference vegetation index (NDVI) provide a measure of greenery within an area as a proportion and a density of vegetation, respectively [40]. These satellite imagery or aerial photography are commonly used as an objective index of greenspace coverage surrounding the individual’s place of residence [47]. Individuals with greater greenspace exposure in terms of higher NDVI values report less perceived stress [33], lower levels of depression [6] and anxiety [12], and healthier cortisol profiles [36]. However, the literature is inconsistent, particularly among older adults. For example, both Astell-Burt et al. [4] and Banay et al. [6] reported statistically significant negative associations between greenspace exposure and both depression and anxiety among older adults, whereas Lee and Lee [26], Pun et al. [33], and Zhou et al. [50] failed to find significant associations between greenspace and mental health.

Although it has not been studied extensively, bluespace (which refers to bodies of water) may also be associated with better mental health [41]. Pearson et al. [31] investigated associations between hospitalizations for anxiety/mood disorder in Michigan and proximity to the North American Great Lakes. They found that anxiety/mood disorder hospitalizations were associated with the percentage of the area covered by inland lakes but did not find a significant relationship with shortened distances to lakes. Helbich et al. [18] reported a potentially protective impact of more blue spaces in the streetscape on depressive symptoms for older people in China. Gascon et al. [16] investigated the effects of long-term exposure to residential bluespaces on anxiety and depression and intake of related medications. The authors reported no statistically significant associations between bluespace and medication use.

Inconsistent findings concerning the association between mental health variables such as depression and both greenspace and bluespace could be due to methodological differences between studies. One key difference concerns how greenspace/bluespace exposure is quantified. Although these studies all purport to measure residence-based greenspace/bluespace, they have varied in terms of level of measurement precision. Previous studies have differed in terms of the size of the buffer distance around individuals’ residence used in calculating NDVI (in the case of greenspace) or normalized difference water index or distance/proportion occupied by blue space (in the case of bluespace), leading to differences in level of measurement precision. At one extreme, some studies purporting to measure residence-based exposure did not have participants’ exact home address, but instead used a proximal location, such as the geometric center of areal units at which individuals reside. Similarly, greenspace exposure has been assessed at a randomly selected time points (e.g., a summer month or month with minimum missing values) without reference to individuals’ time-activity patterns. Thus, greenspace/bluespace metrics based on approximate location and time of exposure likely increase confidence intervals and decrease the power of studies to detect effects on health [1, 17].

In the present study, we examined whether exposures to greenspace/blue-greenspace are associated with lower levels of depressive symptomatology in older adults, and how these associations might vary as a function of the spatial and temporal resolution of greenspace exposure metrics. Specifically, we quantified monthly and annual average NDVI across various buffer distances (i.e., 250, 500, 1,000, and 2,000 m) surrounding the participants’ residences. Using these temporally and spatially resolved greenspace exposure estimates, we investigated the impact of resolution of greenspace exposure on depressive symptoms.

## Methods

### Study population

Study participants were recruited from the Seoul Veterans Health Service Medical Center in the Republic of Korea between January and December 2021 as part of a larger longitudinal project investigating risk for developing dementia [11]. The inclusion criteria for the parent study were as follows: (1) normal ambulation and communication; (2) no physician-diagnosed depression or antidepressant medications; (3) no serious diseases (e.g., cancer, brain infarction, cerebral hemorrhage); and (4) no movement difficulties. Although a total of 671 individuals aged over 60 years were recruited, 54 had missing data on the depression measure and therefore were not included in analyses leaving a final sample of 617 individuals (46.19% female). We collected participants’ residential addresses, which were geocoded using geocoding API of Google Maps Platform (https://developers.google.com/maps/documentation/geocoding/).

### Blue-Greenspace and Greenspace exposure

Highly detailed land use and land cover maps (1 × 1 m resolutions) were used to quantify blue-greenspace exposure. We obtained the latest (updated in 2021) land use and land cover maps from the Korean Ministry of Environment (https://egis.me.go.kr/intro/land.do) that are developed using both satellite images and aerial photography. The blue-greenspace metric was quantified by calculating proportions of blue-greenspace that are associated with specific land use and land cover classes within different buffer distances centered at each participant’s home address. Such land use and land cover classes include public parks, forests, agriculture, and water body. The full details can be found in the Appendix. The ranges of blue-greenspace metric based on land use and land cover maps are between 0 and 1, where 0 indicates no blue or greenspace within the buffer distance and 1 being full of the specific classes within a buffer.

Although the land use and land cover-based metric provides detailed spatial variation of blue-greenspace exposure, it lacks temporal variation. Therefore, we also operationalized exposure to greenery (i.e., greenspace exposure) by calculating the average density of green vegetation within a circular buffer around each participant’s residential address from the surface of NDVI. NDVI values can range between – 1 and 1, with higher numbers indicating a higher density of green vegetation and negative values represents the absence of vegetation [22, 25, 38, 46]. The NDVI data were obtained from the Moderate Resolution Imaging Spectroradiometer (MODIS)/The Visible Infrared Imaging Radiometer Suite (VIIRS) Land Product Subsets project (https://modis.ornl.gov/globalsubset/). Specifically, we extracted vegetation indices that were calculated from global MOD13Q1 data that are provided every 16 days at 250 m spatial resolution between January 1, 2021, and December 31, 2021. On average, two data points were available for each month, and we averaged them to obtain an annual average and monthly average of vegetation indices, respectively. Further we rescaled the NDVI values to 0–1 so that non-vegetated or surfaces with very low-vegetation like river and lake, bare soil, buildings and pavements, or areas covered with snow or ice are represented by a zero value.

For both blue-greenspace and greenspace exposure assessment, we used GIS circular buffers with radii of 250, 500, 1,000, and 2,000 m centered at one’s home address. If the address was near the center of the cell, the selected buffer sizes cover a single cell, the first-order and the second-order neighbors of the overlapped NDVI images. At each location, the NDVI values were summarized as an arithmetic average of NDVI values within the buffer.

### Center for Epidemiological Studies Depression Scale (CES-D-10)

Depressive symptoms were measured using the 10-item Center for Epidemiological Studies Depression Scale (CES-D-10), which is an abbreviated and simplified version of the 20-item CES-D scale [2]. The measure has been validated for older populations [19], has good psychometric properties in both healthy and psychiatric populations [7, 8], and is one of the most widely used screening tools for assessing depression risk in population-based surveys and primary care settings [2]. There are eight negative affect statements (e.g., “I felt lonely”) and two positive affect statements (e.g., “I was happy).” Items are rated based on the past week and the two positive two items are reversed scored. In this modified version of the CES-D, each individual item is rated as “present” or “absent” and total scores can range from 0 to 10. Higher scores suggest greater severity of symptoms, where a cutoff of 4 is defined as having depressive symptoms [19].

### Covariates

Demographic variables consisted of age, sex (male or female), monthly household income (< 1 M, 1 M ~ 2 M, 2 M ~ 3 M, 3 M ~ 5 M, or ≥ 5 M in Korean Won), marital status (married, widowed, divorced, or never married), and education (less than a high school diploma or more than a high school diploma). Participants also self-reported smoking status (current smoker, ever smoker, or never smoked), alcohol consumption (drinker or non-drinker), and physical activity (“During the last 7 days, on how many days were you engaged in moderate physical activity?”). Chronic medical conditions were assessed based on the presence of physician-diagnosed hypertension or dyslipidemia. We further included covariates on environment characteristics, for example annual crime rate per 1,000 people, per capita public park area, and population density, and annual mean temperature, which affect directly or indirectly affect mental health [21, 43, 44].

### Statistical analysis

We hypothesized that both greenspace and blue-greenspace metrics quantified across a wide range of buffer distances (i.e., 250 m, 500 m, 1,000 m, and 2,000 m) have differential effects on depressive symptoms among the elderly. To test this hypothesis, we conducted simple and multiple logistic regression analyses using the presence of depressive symptoms (CES-D cutoff ≥ 4) as the dependent variable and greenspace and blue-greenspace at different buffer sizes as predictors. Here, a separate model was conducted for each buffer size. For blue-greenspace metrics, we used time-invariant but spatially resolved blue- greenspace land use/land cover proportions across different buffer distances. Multiple logistic regression models were adjusted for relevant covariates, including age, sex, household income, education, marital status, current smoking, alcohol status, physical activity, medical conditions, mean temperature, crime rate per 1000 people, population density, and per capita park area. Estimates were presented as odds ratio (OR) and 95% confidence interval (95% CI) of depressive symptoms for an interquartile range (IQR) increase in blue-greenspace and greenspace within 250, 500, 1,000, and 2,000 m centered at one’s home address. In addition, sensitivity analysis was conducted to examine whether individuals’ income modifies the association between greenspace exposure and depressive symptoms by stratifying monthly household income. The low-income group included individuals with monthly household incomes of less than 2 million won, and the high-income group included individuals with monthly household incomes of more than 2 million won. All analyses were performed using Statistical Analysis System version 9.2 (SAS Institute, Cary, NC, United States) and statistical significance was set at p-value (p) < 0.05.

## Results

As seen in Table 1, the sample (*n* = 617; 46.2% female) had a mean age of 73.4 years, with the highest proportion of participants being in their 70s. The proportion of individuals who were non-smokers, non-alcohol drinkers, or had no physical activity was higher than that of their counterparts. More than half of the participants held education levels beyond a high school diploma. The proportion of individuals who were married was the highest at 83.5%. Additionally, over half of the participants had hypertension (54.94%) or dyslipidemia (54.78%), and 23.01% had diabetes. The average population density was 8711.38 people per km^2^ and the crime rates was 26.93 incidents per 1000 people. The average of public park areas per person was 8.27 m^2^. The mean annual temperature was 13.14 degrees.

**Table 1:** Characteristics of the study participants (*n* = 617)

	*N* (Mean)	% (SD)
Age group (in year)		
60–64	34	(5.5)
65–69	77	(12.5)
70–74	249	(40.4)
75–79	202	(32.7)
80–84	55	(8.9)
Sex		
Female	285	(46.2)
Male	332	(53.8)
Household income (in KRW)		
< 1 million	140	(22.7)
1 M ~ 2 M	160	(25.9)
2 M ~ 3 M	128	(20.8)
3 M ~ 5 M	129	(20.9)
≥ 5 M	60	(9.7)
Education		
Less than High School	296	(48.0)
More than High School diploma	321	(52.0)
Marital status		
Married	515	(83.5)
Widowed	89	(14.4)
Divorced	12	(1.9)
Never married	1	(0.2)
Smoking		
Yes	236	(38.3)
No	381	(61.8)
Alcohol drinking		
Yes	217	(35.2)
No	400	(64.8)
Physical activity		
Yes	247	(41.8)
No	370	(58.2)
Medical conditions		
Hypertension	339	(54.9)
Dyslipidemia	338	(54.8)
Diabetes	142	(23.0)
Population density per km^2^	8711.38	(7169.0)
Crime rate per 1000 people	26.93	(1.7)
Park area per person (m^2^)	8.27	(0.6)
Mean annual temperature (degree)	13.14	(0.7)

**Fig. 1:**
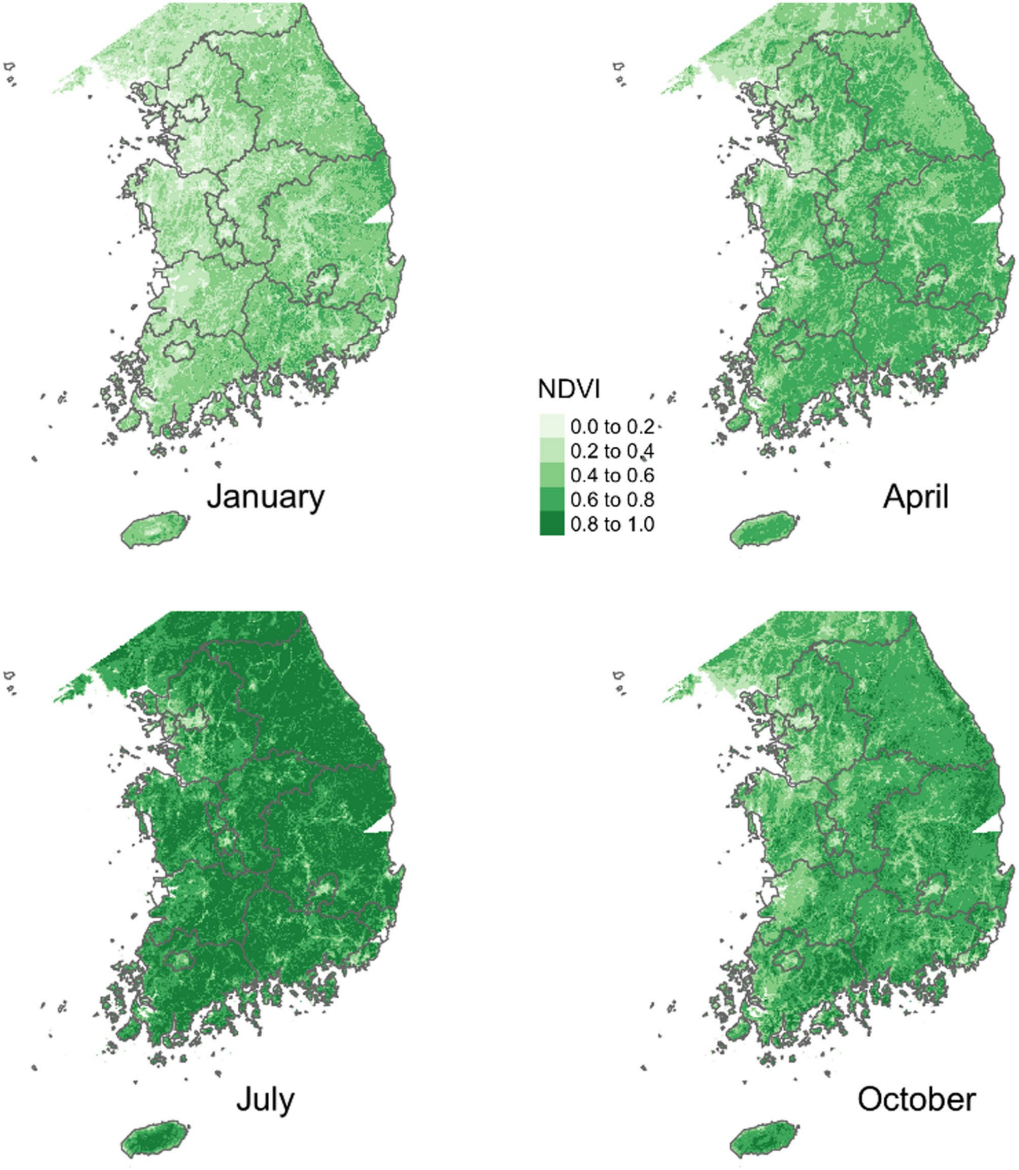
Seasonal variations of NDVI values in South Korea (2021)

**Fig. 2:**
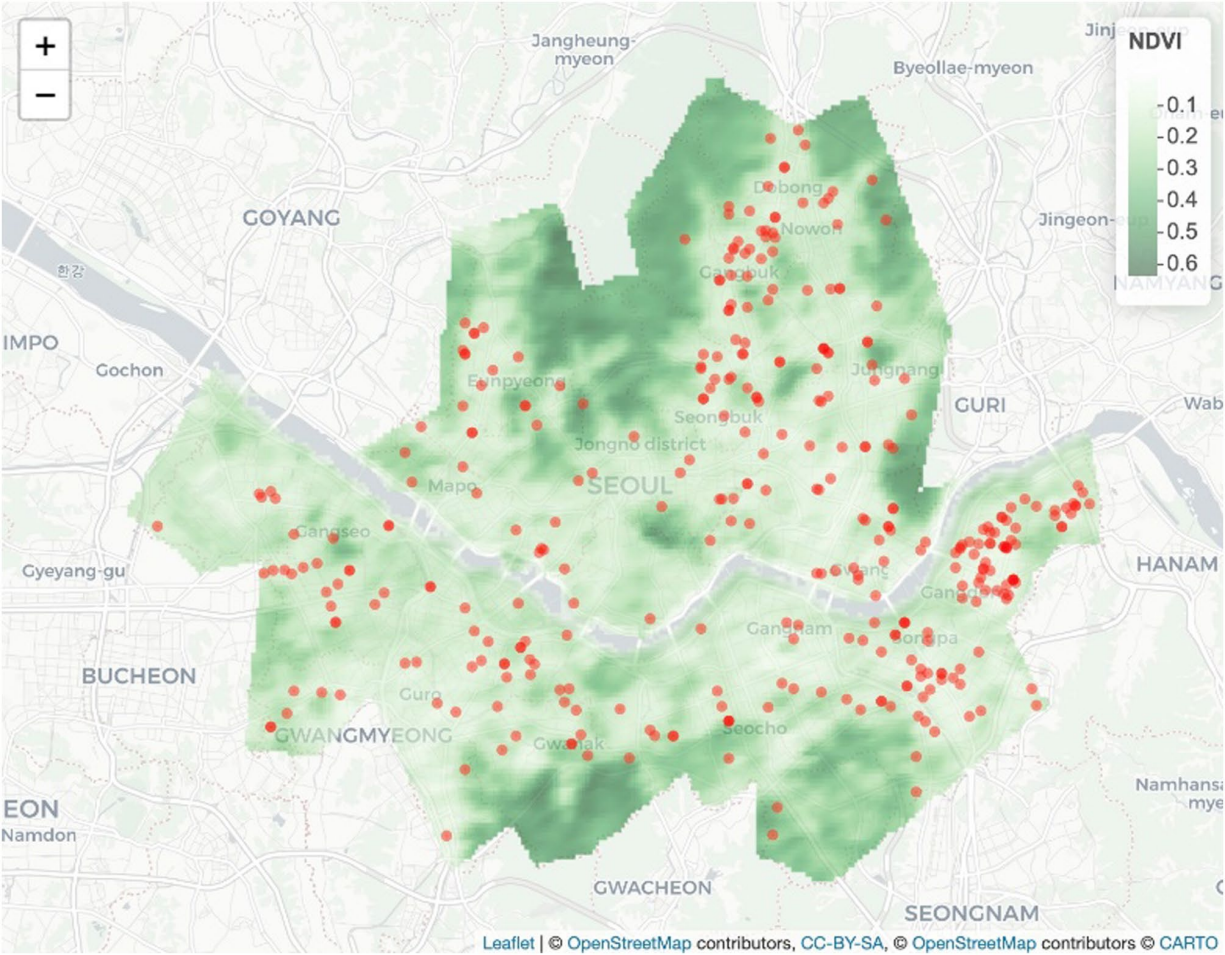
The residence of study participants (denoted as red dots) over the annual average of NDVI values in Seoul, South Korea

The spatial distribution of monthly NDVI values in Fig. 1 illustrates the seasonal variation of greenspace in South Korea. The sparse greenery in winter, quantified by relatively lower NDVI values during January (see the top left panel of Fig. 1), contrasts the lush greenery of middle of summer, quantified by high NDVI values during July (see the bottom left panel of Fig. 1). Annual greenspace exposures were estimated by overlaying the home addresses of the study participants over both the land use and land cover map and the yearly averaged NDVI surface, respectively. The residences of 346 study participants (about 53% of total sample) who resided in Seoul, the capital city of South Korea, are illustrated in Fig. 2 (see the red dots for the location of study participants’ residence).

Table 2 presents the summary of regression model assessing the association between greenspace exposures (quantified as NDVI_250m_, NDVI_500m_, NDVI_1,000 m_, and NDVI_2,000 m_) and the presence of depressive symptoms. In the logistic regression analysis, an IQR increase in the annual average of greenspace within 250 m to 2000 m buffer distances was significantly associated with lower odds of depressive symptoms (Adjusted OR = 0.67; 95% CI, 0.49 ~ 0.91 for NDVI_250m_, Adjusted OR = 0.67; 95% CI, 0.49 ~ 0.92 for NDVI_500m_, Adjusted OR = 0.72; 95% CI, 0.53 ~ 0.97 for NDVI_1,000 m,_ and Adjusted OR = 0.73; 95% CI, 0.55 ~ 0.97 for NDVI_2,000 m_), after adjusting for socio-demographic characteristics, health behaviors, medical conditions, and environment factors. In addition, an IQR increase in blue-greenspace exposures at 250 m and 500 m buffers was significantly associated with lower odds of depressive symptoms (Adjusted OR = 0.59; 95% CI, 0.41 ~ 0.84 for NDVI_250m_ and Adjusted OR = 0.70; 95% CI, 0.52 ~ 0.95 for NDVI_500m_), while no association was observed between blue-greenspace exposure the presence of depression at the other buffer distances.

**Table 2:** Results of logistic regression between annual average of greenspace, blue-greenspace exposure, and depressive symptoms

NDVI	Unadjusted model				Adjusted model*		
	OR(95%CI)		*p*-value		OR(95%CI)		*p*-value
***Greenspace***							
NDVI _250 m_	0.75 (0.57–0.99)		0.0383		0.67 (0.49–0.91)		0.0119
NDVI _500 m_	0.77 (0.58–1.01)		0.0605		0.67 (0.49–0.92)		0.0131
NDVI _1,000 m_	0.81 (0.63–1.05)		0.1127		0.72 (0.53–0.97)		0.0296
NDVI _2,000 m_	0.83 (0.65–1.05)		0.1119		0.73 (0.55–0.97)		0.0321
***Blue-greenspace***							
Blue-green _250 m_	0.65 (0.48–0.90)		0.0082		0.59 (0.41–0.84)		0.0047
Blue-green _500 m_	0.76 (0.58–0.99)		0.0460		0.70 (0.52–0.95)		0.0237
Blue-green _1,000 m_	0.83 (0.63–1.09)		0.1759		0.80 (0.58–1.09)		0.1610
Blue-green _2,000 m_	0.89 (0.68–1.17)		0.4119		0.87 (0.62–1.22)		0.4146

*Adjusted by age group, sex, income, education, marital status, current smoking, alcohol status, physical activity, medical conditions, temperature, crime rate, population density, and per capita park area

We conducted sensitivity analyses by stratifying household income levels and Table 3 shows the associations between both greenspace and blue-greenspace exposure and depressive symptom. In the low-income group, greenspace exposures at 250 m and 500 m were significantly associated with depressive symptoms (Adjusted OR = 0.50; 95% CI, 0.28 ~ 0.90 for NDVI_250m_ and Adjusted OR = 0.54; 95% CI, 0.30 ~ 0.98 for NDVI_500m_). Blue-greenspace exposures at 250 m and 500 m were also significantly associated with depressive symptoms (Adjusted OR = 0.44; 95% CI, 0.23 ~ 0.82 for NDVI_250m_ and Adjusted OR = 0.55; 95% CI, 0.32 ~ 0.95 for NDVI_500m_). Conversely, for the high-income group, no association was observed between greenspace and blue-greenspace exposure and depression at the other buffer distances.

**Table 3:** Results of logistic regression between annual average of greenspace, blue-greenspace exposure, and depressive symptoms, stratified by household income

NDVI	Low-income group			High-income group		
	OR*	(95%CI)	*p*-value	OR*	(95%CI)	*p*-value
***Greenspace***						
NDVI _250 m_	0.50	(0.28–0.90)	0.0207	0.72	(0.35–1.48)	0.3725
NDVI _500 m_	0.54	(0.30–0.98)	0.0433	0.66	(0.31–1.37)	0.2594
NDVI _1,000 m_	0.66	(0.39–1.14)	0.1380	0.68	(0.33–1.39)	0.2912
NDVI _2,000 m_	0.77	(0.48–1.25)	0.2917	0.62	(0.31–1.25)	0.1808
***Blue-greenspace***						
Blue-green _250 m_	0.44	(0.23–0.82)	0.0094	0.70	(0.32–1.53)	0.3718
Blue-green _500 m_	0.55	(0.32–0.95)	0.0330	0.75	(0.37–1.50)	0.4119
Blue-green _1,000 m_	0.68	(0.39–1.19)	0.1740	0.68	(0.32–1.47)	0.3267
Blue-green _2,000 m_	0.80	(0.45–1.43)	0.4529	0.59	(0.25–1.39)	0.2280

Low income group: individuals with monthly household income of less than 2 million won; High income group: individuals with monthly household income of more than 2 million won

*Adjusted by age group, sex, income, education, marital status, current smoking, alcohol status, medical conditions, temperature, crime rate, population density, and per capita park area

**Fig. 3:**
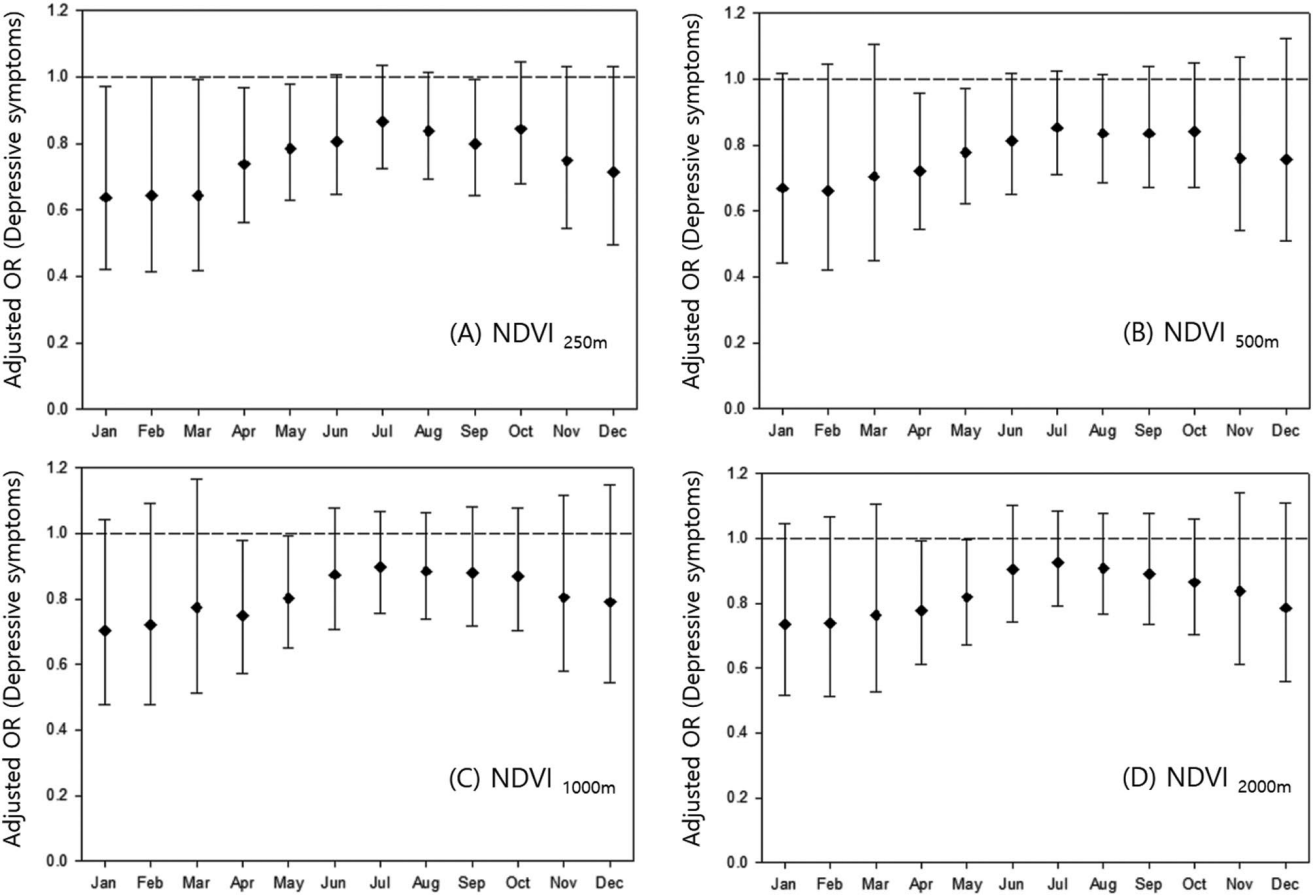
Monthly association between greenspace and depressive symptoms. The model was adjusted for age group, sex, income, education, marital status, current smoking, alcohol status, medical conditions, temperature, crime rate, population density, and per capita park area. The black square and the horizontal line indicate the beta coefficient and the 95% CI, respectively

We repeated the statistical analyses with monthly average of NDVI and Fig. 3 shows the associations between greenspace exposure with 250 m, 500 m, 1,000 m, and 2,000 m buffer distance and depressive symptoms. Overall, the monthly distribution of the association between greenspace and depressive symptoms showed an inverted U-shape; greenness in the winter months was associated with lower odds of depressive symptoms. The odds of depressive symptoms were the lowest for Greenspace during cold months (i.e., January, February, and March) at a 250 m buffer. In NDVI_250m_, depressive symptoms were associated with an IQR increase in greenspace in January, February, March, April, May, and September. In NDVI_500m_, NDVI_1,000 m_, and NDVI_2,000 m_, depressive symptoms were only associated with greenspace in the warm months of April and May.

## Discussion

The present study investigated the association between exposure to greenspace and blue-greenspace and the presence of depressive symptoms among older adults. We found that exposure to both greenspace and blue-greenspace, measured within a short distance buffer (i.e., 250 m) surrounding study participant’s home address, was strongly associated with lower self-reported depressive symptomology among older adults. This association remained significant even after accounting for potential confounding variables, including age, sex, household income, education, marital status, smoking status, alcohol consumption, physical activity, chronic medical conditions, temperature, crime rate, population density, and per capita park area. As such, our results raise the possibility that the presence of greenspace nearby participant’s home address provides protection against depressive symptoms among older adults, particularly in low-income individuals. Perhaps more interestingly, our investigation examined temporal variations in the association between depression and greenspace exposure and found that these were more pronounced during the winter months, particularly at small buffer distances (i.e., 250 m buffer distance).

Despite numerous studies investigating the association between residential greenness and mental health outcomes [4, 10, 14, 15, 23], only a few studies have examined the association between greenspace and depression among older adults, and their findings were inconsistent. Several studies have reported statistically significant negative associations [32], while others reported non-significant associations [26, 33]. In terms of the former, Perrino et al. [32] reported that among US Medicare beneficiaries aged 65 years or older, the odds of depression diagnosis was 8% lower in the medium versus low tertile of greenness (OR = 0.92; 95% CI, 0.88 ~ 0.96), and 16% lower in the high versus low tertile of greenness (OR = 0.84; 95% CI, 0.79 ~ 0.88), independent of individual-level sociodemographic variables [32]. In a UK study, Wu et al. [45] reported that among the elderly living in the highest quartile of greenery had nearly 40% (OR = 0.66; 95% CI, 0.46 ~ 0.95) reduced odds in depression and anxiety compared to those living in the lowest quartile [45]. In a sample of Italian older adults, Ricciardi et al. [34] observed that the exposure to greenspace (quantified by the frequency of greenspace visits and time spent in greenspace weekly) was inversely associated with geriatric depression, and this association was mediated by perceived social support [34]. Banay et al. [6] examined residential greenness within 250-m and 1,250-m buffer distance surrounding the participants’ residences and depression risk prospectively in the Nurses’ Health Study. The authors reported beneficial effects of greenspace within a 250 m buffer on depression among older women (Hazard ratio = 0.87; 95% CI, 0.78 ~ 0.98) [6]. Consequently, the results of primary studies are consistent with ours—that is, the higher the greenspace, the lower the odds of geriatric depression. However, comparing the observed results directly with ours is difficult because of different greenspace assessments (i.e., mean NDVI at 250 m, 500 m, 1000 m, and 2000 m buffer distance vs. mean census block-level NDVI vs. the amount of greenspace and private gardens in the geographical unit) and study designs (i.e., cross-sectional study vs. prospective cohort study).

We also found that lower income levels increased the protective association between greenspace exposure and depressive symptoms among older adults. Although no study shows the role of older people’s income in modifying the observed association, previous studies have suggested that greenspace contributes to better health outcomes for vulnerable individuals [29, 35, 49]. Our results are consistent with the notion that low-income people could be particularly benefit from more green environments.

Our analyses suggest that there are positive effects of residential greenspace and blue-greenspace exposure on depression among the elderly, but that this effect may be limited to greenery in the immediate area surrounding the person’s residence. The present study makes a unique contribution to the literature by examining the sensitivity of buffer distance in greenspace exposure on the impact of residential greenery on depression among elderly populations. We found that greenspace and blue-greenspace exposure within 250 m around each participant’s home address was most strongly associated with fewer depressive symptoms. A growing body of studies on GIS buffer analyses also considered how the buffer distance within which greenness is measured can have a major influence on whether studies can detect associations between greenspace and health outcomes [9]. Although greenspace studies on depression among elderly populations are limited, Banay et al., [6] found that living in the highest quintile of residential greenness assessed within a 250 m buffer had significantly reduced risk of developing depression compared to those living in the lowest quintile. In contrast, in this same study greenspace assessed within a 1,250 m buffer was not statistically significant. Due to reduced mobility, older individuals may find nearby greenery particularly important, though see Pun et al. [33] for conflicting results [33]. Interestingly, several past studies found that greenspace exposure is associated with lower depression among older individuals who are more active [3, 5, 39], when exposure estimates are based on actual time spent in parks, and when the buffer is relatively small (at the census tract or census block level).

In the present study, the 250 m buffer distance was intended to reflect the more immediate visual environment around participants’ residence, whereas the 2,000 m buffer was intended to reflect the higher end of the distance range study participants may be willing to walk from their homes to an environmental feature [20]. While more greenspace may promote physical health by providing opportunities for physical activity [42], it may be that older individuals in general tend to benefit more from the greenery in the immediate visual environment. Consequently, further research on mental health findings related to greenspace at different distances is needed to expand the knowledge on the effects of greenspace on mental health in older adults.

In addition to the issue of spatial resolution, our study demonstrated that the dynamic nature of the association between depression and greenspace, which varied over the 12-month period. Most studies limit data observation to the late spring, summer, and early fall months (at least in temperate regions) based on the assumption that greenspace will only impact health and mental health during these seasons. However, to our knowledge this assumption has never been empirically tested. In contrast, our results indicate that greenspace was most strongly associated with depressive symptoms (CES-D < 4) during the winter months (i.e., January, February, and March). In part this result may be due to South Korea’s forests consisting mainly of coniferous trees, which typically do not lose their leaves in winter [24]. However, it may also be in part due to the differences in amount of residential greenery having a stronger impact on behavior and functioning when overall greenery is limited. For example, perhaps having relatively more residential greenery in the winter motivates individuals to be physically active during the darker and colder days of winter when they otherwise would be inactive.

Only a few studies have highlighted seasonal differences in greenspace exposure on physical exercise [37, 50]. For example, Shin et al. [37] examined greenspace data estimated by both the maximum of multiple NDVI values during the study period, which likely only included summer months, and the yearly averaged NDVI values, which included both summer and winter months [37]. They found that the former was not significantly associated with exercise, whereas the latter was positively associated with exercise (*p* < 0.01). These results are consistent with our post-hoc hypothesis about the heightened impact of wintertime greenspace exposure on physical activity [37]. In contrast, Zhou et al. [50] found that greenspace exposure assessed during the summer promoted physical activity more strongly compared to winter greenspace exposure. Given these mixed findings, further research is necessary [50]. The strength of this study is novel finding on spatiotemporal variability of the association between greenspace exposure and depressive symptoms in older adults. Although researchers have paid much attention to greenspace as an environmental component in reducing the risks of mental disorders, studies on the effect of greenspace exposure on older adults’ depression is rare. In addition, although the availability of satellite imagery at a fine spatial resolution for calculating greenness has been enabled, the optimal buffer distance and time for greenspace exposure on mental health benefits still need to be discovered. The positive association between greenspace near their home address (i.e., as close as 250 m) or in the winter months and depression in later life would be a notable new outcome. However, the present study has several limitations. First, the analysis is cross-sectional, meaning a causal relationship between depressive symptoms and greenspace cannot be established. Second, depression was defined using the CES-D, a frequently used screening instrument for mental disorders and depression in older adults [7, 8]. However, CES-D is not a diagnostic tool, and thus it may not reflect clinician-diagnosed depression. Also due to the exclusion criteria of the parent study, individuals who had been diagnosed as having a depressive disorder by their physician were not included in this study. Consequently, our sample likely had less severe symptomatology and less variance in depression than the general population, reducing our power to detect associations. Third, NDVI is a valid and objective measure of greenspace, but it cannot distinguish the types and quality of green areas and the usability of greenspace, which may influence mental health. To explore the spatial effect of different buffer distances in greenspace exposure on depression, more analysis is needed on the spatial heterogeneity analysis across broader geographic regions in South Korea. However, since 93% of study participants are concentrated in two provinces (Seoul: 53% and Gyeonggi: 40%), our sample simply does not have sufficient spatial heterogeneity for such an analysis. Furthermore, NDVI provides a top-down view of vegetation instead of a ground-level, human perspective of greenspace. Alternatively, green view index (GVI), based on images captured from Google Street View along the street around study participant’s home address, may provide pedestrians’ view of greenspace and their experience in urban environment [27]. In addition, because we calculated the amount of greenspace as the NDVI around participants’ homes, it did not reflect dynamic natural environmental exposures while they are likely exposed during their routine activities occurring outside of their homes. For example, some may have had relatively little greenery around their home but spent considerable time visiting friends in greener neighborhoods. The limited scope of exposure assessments may increase exposure misclassification and affect health-effect estimates. Fourth, we could not rule out the likelihood of unmeasured and residual confounding variables (i.e., patterns of availability, accessibility, and uses of green spaces) being associated with the exposure and outcomes. Finally, although the residences of the study sample were generally distributed throughout Seoul (Fig. 2), they were not randomly assigned and were only recruited from one medical outpatient department. The findings may thus not be generalizable to all circumstances in older adults.

## Conclusions

We found that older adults living around urban greenspace had fewer symptoms or lower rates of depression. The observed association varied based on spatial resolution and temporal dynamics; greenspace within 250 m of their home or in the winter months were more strongly associated with lower depression than at greater buffers and during other seasons of the year. Our findings suggest that higher levels of neighborhood greenspace may be a protective factor against depression in older adults, but that the benefits may depend on the spatial and monthly distribution of greenspace.

## Electronic supplementary material

Below is the link to the electronic supplementary material.

Supplementary Material 1

## Data Availability

The data that support the findings of this study are available from the corresponding author upon reasonable request.

## Abbreviations

CES-D: Center for Epidemiological Studies Depression Scale
MODIS: Moderate Resolution Imaging Spectroradiometer
NDVI: normalized difference vegetation index
SE: standard errors
