# Supplementary material for: Spatiotemporal variability of the association between greenspace exposure and depression in older adults in South Korea
Source: BMC Public Health. 2024 Sep 19;24:2556. doi: 10.1186/s12889-024-19952-2 (PMC11414157; doi:10.1186/s12889-024-19952-2)
Supplement: Supplementary file 1 — Supplementary Material 1 [file 12889_2024_19952_MOESM1_ESM.docx]

1. Land cover/land use classification used for blue-greenspace

We found a total of 41 detailed land cover classes in the Korean land cover mapping system (<https://egis.me.go.kr/intro/land.do>). We calculated the blue-greespace based on 25 classes the culture/exercise/recreational facilities (141), agriculture (211, 212, 221, 222, 231, 241, 251, 252), forest (311, 321, 331), grassland (411, 421, 422, 423), wetland (511, 521, 522), vacant land (611, 612, 613), and water body (711, 712, 721). The numerical number refers to the classification code.
